# Factors in consumers' purchase intention for Gejia batik

**DOI:** 10.1016/j.heliyon.2023.e23085

**Published:** 2023-11-30

**Authors:** Xizhen Li, Nurul Hanim Romainoor, Zhiqin Sun

**Affiliations:** a School of Design and Art, Yancheng Institute of Technology, Yancheng City, 224001, China; b School of the Arts, Universiti Sains Malaysia, Penang, 11800, Malaysia

**Keywords:** Intangible cultural heritage, Gejia batik, Purchase intention, Qualia, Attitude, Product

## Abstract

In China's vigorous development and inheritance of intangible cultural heritage, the sustainability and acceptability of intangible cultural heritage products have become a controversial subject. This study aims to explore the relationship between batik product qualia factors and the purchase intention for batik products and exammine the mediating role of consumer attitude in the relationship. We adopted quantitative research methods and used SPSS 26 and Process 2.15 software to test our hypotheses. We conducted extensive surveys of consumers of different ages, genders, income levels, and educational backgrounds, and finally, a total of 381 valid questionnaires were collected. The results showed that batik products' creativity, delicacy, beauty, and eco-friendliness were significantly and positively related to consumers' attitudes. In addition, creativity, beauty, and eco-friendliness, but not delicacy, were significantly and positively associated with consumers' purchase intention. Consumer attitude plays an intermediary role between qualia factors and purchase intention. This study analyzes Gejia batik from the perspective of qualia factors, breaking through the limitations of previous studies on the aspects of heritage protection and environmental protection. The study's results can inspire batik manufacturers or designers to enhance the competitiveness of batik design products in the tourism market.

## 1 Introduction

According to United Nations Educational, Scientific and Cultural Organization's (UNESCO's) World Heritage Convention, heritage is invaluable and irreplaceable for every country and all of humanity, as once it is lost, it is difficult to restore. Intangible cultural heritage (ICH) represents a country's image and serves as the foundation of national identity. ICH refers to the oral traditions, festive activities, rituals, and performing arts that have been passed down through generations and are considered by communities as a part of their cultural heritage [1]. However, with the development of the economy and society, ICH faces the risk of disappearance in many regions. The high cost and time-consuming nature of producing ICH due to its purely handmade nature have resulted in high prices. Additionally, the styles of ICH often do not align with modern trends, which has contributed to the relatively low popularity of ICH products (ICHPs) among modern people. However, with the establishment of the UNESCO Intangible Cultural Heritage Lists and increasing worldwide recognition and government support, ICH has experienced significant revitalization [2]. Some governments have begun to see ICH as a source of income, and many regions have designed ICHPs in the name of preservation [2]. Nevertheless, many designs of ICHPs are too traditional and do not meet modern people's aesthetic demands. Therefore, how to design ICHPs that meet modern people's needs is an essential aspect of achieving the sustainable development of ICH. This study addresses this issue by proposing a conceptual framework to investigate consumers' purchase intentions.

Gejia batik is an ICH in Southeast Guizhou, China. It was included in the third batch of the national-level ICH list in 2011. The production process for Gejia batik comprises mainly five steps: making a design on a cloth; attaching wax onto the pattern; the dying and fixation process; the wax-removal process; and washing the cloth and drying. Gejia batik is considered a representative feature of the local culture. In Huangping, Guizhou, the sales of Gejia batik are managed by local inheritors of ICH. However, due to the lack of understanding of modern people's consumption demands by local inheritors, the development and dissemination of Gejia batik products have stagnated. In the dissemination and preservation of ICH, many studies have been conducted from the perspective of residents or local governments [[3], [4], [5], [6], [7]]. Few people have paid attention to the perspective of tourist consumers. However, ICHPs can only increase income and employment opportunities for local people and achieve the sustainable development of ICH if they are recognized and liked by tourist consumers. In recent years, most studies on batik have focused on environmental protection, mainly on the wastewater-treatment strategy of batik [8], application of natural dyes [9,10], and how to improve the ecological problems in the batik-production process [11]. Some studies focus on the purchase decision for batik products in Indonesia and Malaysia. Some scholars have conducted comparative studies on the factors in the purchase of batik by Indonesian and Malaysian consumers, mainly through an analysis of perceived value and quality [12]. However, few studies focus on the purchasing intention for Gejia batik in China.

Furthermore, researchers have yet to examine Gejia batik from the perspective of the qualia aspect. Therefore, this study attempts to fill this gap by analyzing factors associated with Chinese consumers' purchase intention for Gejia batik. This study focuses on three research questions: What factors relate to consumers' purchase intention for Gejia batik? How are these factors associated with consumers' willingness to buy Gejia batik? Which factors have the strongest relationship? Thus, this study considers Gejia batik as an ICHP, explores the relationship between qualia factors and consumers' purchase intention, such as beauty, creativity, delicacy, and eco-friendliness, and further examines how these factors are related to consumers' purchase intention.

Our research offers three significant contributions to the sustainable development of ICH. First, it pioneers academic research in a product category that is highly significant in the consumer domain but has long been overlooked – ICHPs. These products bear witness to the importance of human culture and can only thrive in today's society if consumers love and appreciate them. Second, by exploring the factors associated with consumers' purchase intention for batik, we can provide design guidance for batik designers and inheritors of ICH. Third, understanding the mechanisms associated with consumers' purchase intention helps local governments make policy decisions and enhance the development of the local tourism economy.

We first introduce the key concepts related to the purchase-decision process for batik products. We then develop the research hypotheses. We next use statistical software to test the hypotheses. Last, we discuss the research findings, the theoretical contributions, and managerial implications for both batik designers and decision makers.

## 2 Literature review

### 2.1 Qualia factors

In recent years, with the rapid development of the economy, people's demand for products has extended beyond functionality to include beauty, creativity, delicacy, and other emotional attributes. These qualia factors have become crucial factors for consumers in purchasing a product. Lewi [13] was the first to use the term “qualia” in its modern sense. Qualia are the most fundamental instincts of human cognition, representing our most direct perception [14]. This perception is projected onto tangible objects and can be achieved through the presentation of products or services [15]. In the product-design field, Yen proposed the perceptual evaluation model, suggesting that in order to understand consumers' attitudes and emotions toward products, design should be measured in terms of attractiveness, aesthetics, creativity, delicacy, and engineering. Ashby and Johnson [16] proposed the theory of product personality, which suggests that products have two dimensions: physiological (e.g., features that fulfill the primary purpose of design, functions, and other features) and psychological (e.g., personality and usability). Here, delicacy and engineering are a product's physiological qualities, while attractiveness, aesthetic appeal, and creativity are its psychological qualities. If a product can simultaneously meet both physiological and psychological qualities, it can satisfy consumers in terms of functionality and emotions [17].

Like other products, Gejia batik, as a local tourism product, shares the dual attributes of functionality and emotion. Rahadi's empirical research has found that batik patterns, design, quality, and price play a significant role in consumers' purchasing decisions. Ramlee [18] believes that quality is essential when customers purchase batik products. Simin's [19] research has identified six factors in the purchase of batik products in Malaysia: quality, price, availability, packaging, reference, and brand. Additionally, open-ended questions revealed that other factors, such as design, authenticity, and usability, affected purchasing intention. Paramita's [20] research has demonstrated that the creativity of batik products is significantly related to consumers' repurchase intentions. Therefore, based on the literature review results, this study divides the qualia factors of batik products into four dimensions, considering the unique characteristics of batik products themselves: beauty, creativity, delicacy, and eco-friendliness.

#### 2.1.1 Beauty

Beauty is considered a product's core feature and success factor. Users obtain a sensation of pleasure through the design of the product's appearance. It creates a contextual feeling that allows consumers to experience practicality and evoke positive imagination. The beauty of a product is conveyed through elements such as form, color, proportion, etc., and it triggers a sense of aesthetic perception in consumers' minds through visual perception [21]. Design researchers generally consider a product's beauty from two perspectives: sensory, mainly visual, manifested by the product, and specific cognitive and emotional responses to the product. Thus, this study defines beauty as the aesthetics of product appearance, referring to the objective design qualities presented by Gejia batik products, such as shape, form, and color.

Beauty is among consumers' initial reactions to a product, closely linked to visual information. Therefore, it is significantly associated with the product's overall perception. For products that differentiate themselves from competitors in functionality and pricing, beautifying becomes especially important as it often provides consumers with additional value propositions. Thus, beauty is considered a critical factor in customers' purchase decisions and satisfaction.

#### 2.1.2 Creativity

Creativity is thought to be a fundamental element of human intelligence. In design, researchers have proposed various definitions of creativity [22]. For example, Lee et al. [23] stated that creativity was a measure of value or novelty that is expressed in a design. Valgeirsdottir et al. [24] suggested that creativity was the process of developing novel and original ideas that were adapted to a particular function or occasion in a way that brought value to potential users or adopters. Yen et al. noted that creativity was the ability of a product to provide a differentiated and unique experience. However, the nature of creativity is so complex that there is no single and fixed definition that can accurately capture this concept. Rodgers and Jones [25] indicated that defining creativity challenged design scholars. Therefore, based on the literature review and inherent characteristics of Gejia batik products, the creativity defined in this study refers to the novelty, originality, and ability to generate interest in Gejia batik products.

Creativity plays a significant role in the early stages of design and is beneficial for long-term business performance [26]. It has been widely recognized as an essential component of the ideation phase in the design process, as innovative and successful designs often stem from creative concepts [27]. Therefore, creativity is considered a critical factor in designing and developing new products.

#### 2.1.3 Delicacy

The delicacy of a product is an essential concept in product design and manufacturing. It encompasses a product's refinement and high quality in terms of its appearance, materials, craftsmanship, and details. There are multiple definitions of product delicacy. One standard view is that delicacy refers to a product's elegant, precise, and refined characteristics, reflecting the designer's attention to detail and pursuit of exceptional craftsmanship. Another viewpoint is that delicacy represents the high quality and exceptional craftsmanship exhibited throughout a product's overall design and manufacturing process. Yen et al. [21] noted that delicacy in a product referred to the completeness and meticulous handling of its functionality, which evoked a sense of refinement for consumers. She also maintained that delicacy was closely related to details, and exquisite products required detailed design to highlight their quality. Some scholars argue that delicacy is a physical aspect of a product that can influence people's emotions and attention [28]. This study defines delicacy as the craftsmanship and intricacy of the patterns in batik products. Chuang and Ma [29] point out that a product's level of delicacy and quality can attract consumers and be associated with purchase decisions.

#### 2.1.4 Eco-friendliness

The ecological friendliness of design products is an important aspect in modern design. Faced with severe environmental challenges and the need for sustainable development, an increasing number of designers and manufacturers are starting to pay attention to how to develop and produce environmentally friendly products. An eco-friendly product could be defined as “one constituted of materials and associated with production practices along its entire life cycle recognized for being socially and environmentally responsible” [30]. In product design, researchers have systematically discussed green materials, technologies, and manufacturing [31], as well as other aspects to reduce resource consumption and mitigate negative environmental impacts. In product packaging, many researchers have focused on green packaging, sustainable, ecological, and environmentally conscious designs [32,33]. These studies aim to reduce reliance on non-renewable resources and promote sustainable development. Moreover, studies have shown that consumers' knowledge and attitudes toward green products positively correlate with their purchase intention for eco-friendly batik [34].

According to the batik-production process previously mentioned, the materials used in the dyeing process and wastewater generated during the dyeing and dewaxing process can impact the environment. Thus, the ecological friendliness of batik products mainly focuses on dyes, dyeing processes, wastewater treatment, and environmental pollution [9,35]. However, studies have shown that despite using natural dyes, the wastewater produced by the batik process can still impact our environment [9], and most batik workshops do not have the conditions for special wastewater treatment. Moreover, the impact on the environment caused by the batik production process is only apparent to the producer. Therefore, in this study, consumers' purchase intention is related to their self-perception of whether batik is environmentally friendly.

### 2.2 Attitude

Attitude is considered one of the essential variables for analyzing individual consumer behavior and has been examined in various marketing fields [36]. It reflects an individual's positive or negative beliefs, ultimately influencing their decision to engage or not engage in a particular behavior [37]. According to Ajzen [38], an attitude is a psychological tendency expressed through an individual's favorable or unfavorable evaluation of a specific behavior. Therefore, a person with a positive belief is more likely to intend to carry out the related behavior. Attitude, as a measurable psychological construct, is relatively enduring and stable. It can influence and predict the generation of behavior. Currently, there are numerous studies on attitude prediction and behavioral intentions, predominantly in the field of consumer behavior, which have yielded significant results. For example, Hsu and Chang [39] examined consumer purchase intentions from the food safety perspective. They believed that consumers' attitudes toward food safety were associated with their food acceptance and purchase intentions. Mosunmola et al. [40] pointed out that attitude toward online shopping was significantly and positively associated with the behavioral intention to use online shopping. Vergura et al. [41] examined consumers’ attitudes toward purchase intention for organic personal care products and found that both the affective and the utilitarian attitudes were significantly associated with purchase intention. Thus, attitude is associated with behavioral intentions, and it is included in this study's model.

### 2.3 Purchase intention

The importance of understanding the individual decision-making process has led to a series of studies on the association of purchase intention with consumer behavior. Purchase intention refers to the willingness or inclination that consumers form before engaging in purchasing behavior. According to Ajzen [42], behavior is driven by intentions, which means that a person will engage in particular actions when considering consumption situations. Therefore, intentions serve as indicators of possible future individual purchasing behavior. The higher the likelihood, the stronger the purchase intention. Understanding consumers' purchase intentions is crucial for marketing and product development. It helps companies analyze the market and adjust their products or services to increase sales and profit. Therefore, numerous studies have been devoted to exploring the formation process of purchase intentions and their factors. Modig [43] pointed out that product quality and brand advertising were significantly associated with consumers' purchase intention. Lim [44] examined the factors related to consumers' willingness to shop online, and the results showed that subjective norms and attitudes were significantly associated with the intention to shop online. In addition, Ramadania [45] investigated how consumer ethnocentrism related to consumers' preference for domestic products. The results showed that consumer ethnocentrism was significantly and positively related to consumers' willingness to purchase domestic products, and product attitude played a mediating role in the relationship. Based on the previous study, purchase intention is an essential variable in examining sales processes for Gejia batik products. It is also the ultimate goal that local managers hope to achieve with consumers.

## 3 Materials and methods

### 3.1 Study case and research object

Gejia batik is among the traditional handicrafts in Southeast Guizhou, China. It originates from the wisdom and creativity of the local Gejia people. This traditional batik technique has been inherited and developed in the Gejia villages, becoming a treasure of cultural heritage in the region. Gejia batik is renowned for its exquisite craftsmanship and unique pattern designs. The patterns are diverse and abundant and often originate from folklore, plants, and animals in daily life. They show the local Gejia people's passion for life. These patterns form a unique artistic style and visual charm through batik practitioners' meticulous conceptualization and skillful expression. Gejia batik has been passed down through the generations, carrying the Gejia people's wisdom and emotions. It is not only an artwork but also a cultural heritage and a bond of communication and transmission. By preserving, inheriting, and promoting Gejia batik, we can enable more people to understand and appreciate this precious cultural heritage while promoting the prosperity and development of the local economy.

### 3.2 The selected batik products for testing

The accuracy of social science questionnaire surveys is often influenced by the personal experiences and environments of the respondents. In recent years, batik products have experienced rapid development in southwest China. However, the market size is not significant from an overall industry perspective. Therefore, to control for the potential information bias caused by the lack of cognitive experience with Gejia batik products among the research subjects, this study selected currently available Gejia products in the batik market as the test products.

The selection of test products mainly involved the following steps: first, the initial screening of several renowned villages known for selling Gejia batik products, including the Fengxiang, Wangba, Tangdu, and Matang villages; second, choosing one handcraft workshop that was recognized as an inheritance base by the governments of the four villages; third, selecting the top 10 selling batik products from each of the four handcraft workshops; in total, there were 40 Gejia batik products; fourth, inviting research experts in the field of fashion or textiles and finally selecting 16 batik products as the final test products based on the principle of selecting at least one product from each category and the representativeness of the products and research objectives (Table 1).

**Table 1:**
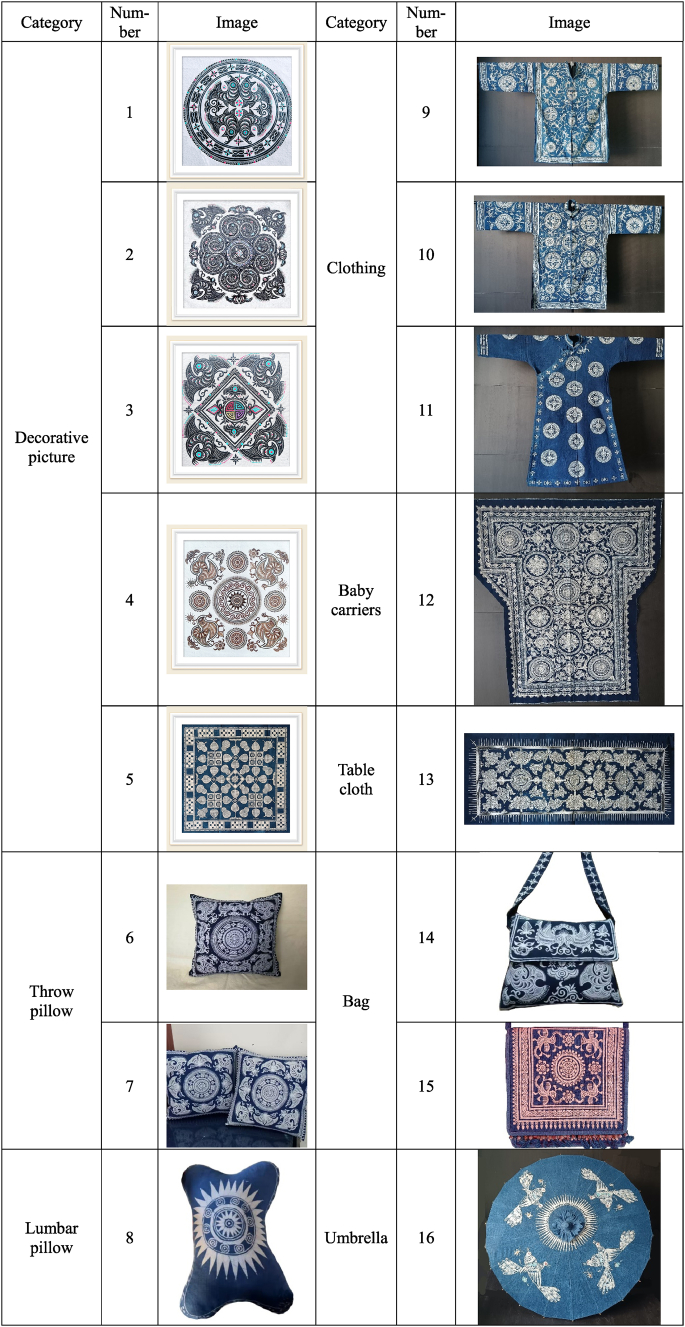
The selected batik-product list.

### 3.3 Questionnaire design

The questionnaire is divided into two parts to collect consumers' thoughts on Gejia batik. The first part comprises respondents' demographic characteristics, including age, gender, education level, and monthly income. The second part constructs three categories of measurement indicators: batik qualia features (including beauty, creativity, delicacy, and eco-friendliness), consumer attitudes, and consumer purchase intentions. Each construct's items were adapted from Yen's [21] and Liu's [46] studies. We invited three experts in the field of study to evaluate the content of the questionnaire to help determine whether the questions were appropriate, the concepts were accurately measured, and the questions were easy to understand. Based on the expert evaluation and feedback, the questionnaire items were revised and improved. Finally, 24 items were extracted, as shown in Table 2. The questionnaire was mainly structured on the path of qualia factors-consumers' attitude-purchase intention, beginning with Gejia batik qualia factors and then exploring the formation mechanism of purchase intention based on respondents' perceptions. When constructing the qualia dimension, we redefined multiple qualia dimensions based on the characteristics of Gejia batik, adding consumers' attitudes as a mediator variable. Items were scored on a 7-point Likert-type scale (1 = strongly disagree; 2 = disagree; 3 = somewhat disagree; 4 = neutral; 5 = somewhat agree; 6 = agree; and 7 = strongly agree).

**Table 2:** Measurement indicators for elements of Gejia batik.

Level 1 Indicator	Level 2 Indicator	Definition of level 3 indicator
Qualia factors	Beauty	This Gejia batik product has aesthetic appeal.
		The color combination of this Gejia batik product is harmonious. This batik product has a beautiful pattern
		This Gejia batik product is visually pleasing.
	Creativity	This Gejia batik is a creative product.
		This Gejia batik product is well-designed This Gejia batik product possesses a certain level of artistic quality.
		This Gejia batik product evokes a sense of novelty
	Delicacy	This Gejia batik product is exceptionally exquisite. This Gejia batik is finely crafted.
		This Gejia batik product has a high level of craftsmanship and quality.
		This Gejia batik was drawn with fine lines.
	Eco-friendliness	I think the fabric of Gejia batik is comfortable, healthy, and consumer-friendly. I think Gejia batik products have a minimal impact on the natural environment during the manufacturing process.
		I believe that the fuel used in the production process of Gejia batik is safe and harmless to human health.
		I believe that Gejia batik is environmentally friendly in terms of the materials used in their production process.
Attitude	Consumers' attitude	I think Gejia batik is excellent. I like Gejia batik products
		I prefer Gejia batik products over other batik products.
		I think Gejia batik products are worth buying.
Intention	Purchase intention	I will consider purchasing Gejia batik products. I am very likely to buy Gejia batik products in the future.
		I will recommend that others purchase Gejia batik products.
		When encountering similar products, I would prioritize considering purchasing Gejia batik products.

### 3.4 Research hypotheses

**Fig. 1:**
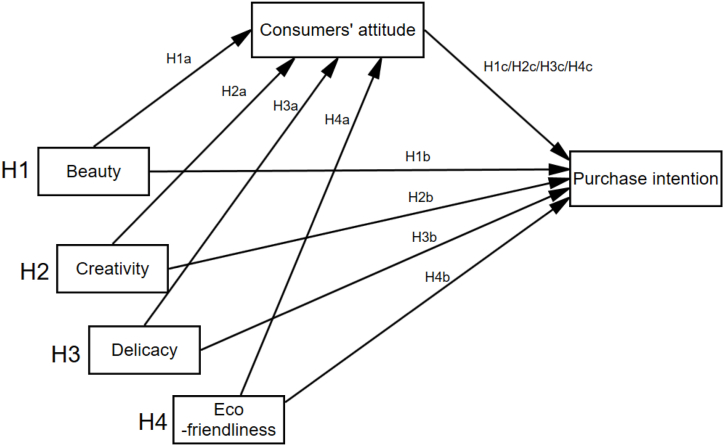
Hypotheses in the proposed qualia factor—consumers’ attitude—purchase intention model.

Based on the literature review and research design, we hypothesized that consumers' perceptions of the qualia of Gejia batik products were positively related to their purchase intentions. Specifically, consumers' attitudes were expected to mediate the relationship between the four qualia perception indicators and consumers' purchase intentions. The assumptions were formulated as follows (Fig. 1).

Hypothesis 1 Beauty is positively and significantly associated with consumers' attitudes (H1a) and purchase intention (H1b); consumers' attitude mediates the relationship between beauty and purchase intention (H1c).

Hypothesis 2 Creativity is positively and significantly associated with consumers' attitudes (H2a) and purchase intention (H2b); consumers' attitude mediates the relationship between creativity and purchase intention (H2c).

Hypothesis 3 Delicacy is positively and significantly associated with consumers' attitude (H3a) and purchase intention (H3b); consumers' attitude mediates the relationship between delicacy and purchase intention (H3c).

Hypothesis 4 Eco-friendliness is positively and significantly associated with consumers' attitude (H4a) and purchase intention (H4b); consumers' attitude mediates the relationship between eco-friendliness and purchase intention (H4c).

## 4 Results

### 4.1 Item analysis

We used SPSS 26 for item analysis to assess whether our proposed scale could differentiate the respondents' extent of attitude toward and purchase intention for Gejia batik. The pre-test sample comprised 58 consumers of different ages, social backgrounds, and cultural levels. We used t-test for item analysis. The scores for all the items in each scale dimension were separately aggregated, and the values corresponding to the 27th and 73rd percentiles were identified. Using the values corresponding to the 27th and 73rd percentiles as benchmarks, we then categorized the total scores for each dimension into low- and high-score groups. Finally, we conducted an independent samples *t*-test to compare the two groups' data. The results of the *t*-test indicate a significant difference in the mean scores between the high and the low-score groups for all the dimensions (p < 0.05) (Appendix Table A). This suggests that all the questionnaire items can differentiate the respondents' extent of the response. Therefore, we deemed the questionnaire suitable for a formal investigation.

### 4.2 Common method bias

Because all the critical variables in this study were generated from self-reporting by individual participants, the relationships between the variables would inevitably be influenced by common-method bias (CMB) [47]. This study adopted two procedural control methods to reduce the potential bias due to CMB. First, CMB was corrected through anonymous testing. Before the survey, the participants were assured that an anonymous testing method would be used to ensure their responses to sensitive questions were not influenced by excessive subjective biases. Second, the measurement order was altered in the questionnaire design by randomly arranging the order of items to disrupt any potential response patterns due to the common measurement method. Although this study employed procedural-control methods to reduce the impact of CMB, it was necessary to conduct statistical tests to examine the potential presence of CMB in the measurement data [47]. We first checked whether our data followed a normal distribution. The absolute values for the skewness and kurtosis for all the variables were less than 2 and 7, respectively (Appendix Table B), indicating that the data did not exhibit severe non-normality. We then conducted confirmatory factor analysis using AMOS v.26. The results showed that the fit of the single-factor model (χ2/df = 2.395, CFI = 0.964, TLI = 0.965, RMSEA = 0.061) was significantly worse than that of the six-factor model (χ2/df = 9.715, CFI = 0.749, TLI = 0.750, RMSEA = 0.151), further confirming that CMB in this study was not severe.

### 4.3 Formal data processing and analysis

#### 4.3.1 Demographic and statistical analysis of respondents

We conducted the formal survey using non-probability sampling (convenience sampling). We selected individuals or subjects who were easily accessible and willing to participate in the study, and all the respondents were from China. For example, we chose university campuses, supermarkets, and commercial plazas with a large flow of people as the main locations for distributing the questionnaire. We also used an incentive mechanism: The respondents willing to participate in the survey would receive a small gift worth approximately 5 yuan. This research lasted almost a half month, from June 8, 2023, to June 26, 2023. During the survey period, 450 questionnaires were sent out, and 412 respondents filled out the questionnaires. However, 31 questionnaires were invalid, 16 filled in the same options, and 15 had missing values. After excluding invalid questionnaires, a total of 381 valid questionnaires were obtained, with an effective response rate of 85 %. The questionnaire recovery rate was consistent with the standard for statistical analysis. Furthermore, we used G*power 3.1.9.7 to calculate the required sample size. We used test family: exact, the statistical test part chooses correlation: bivariate normal model, with a two-tailed alpha value of 0.05, the correlation p H1 at 25, correlation p H0 at 0, and power at 0.95. Finally, the calculated sample size was 202; thus, the 381 valid samples obtained in this study were sufficient. Through descriptive statistical analysis of the demographic variables (Table 3), we found that the proportion of female respondents accounted for 52.8 %, slightly higher than that of male respondents at 47.2 %. According to the data, young and middle-aged adults are the leading demographic group in the sample, with individuals aged 18–29 accounting for 49.1 % and those aged 30–39 accounting for 30.2 %. Most respondents have a relatively low monthly income, with 61.2 % earning less than 5000 yuan. The remaining respondents earn a monthly income greater than 5000 yuan. Most respondents have received a good education, with 40.9 % of the respondents having completed undergraduate studies and 40.9 % of the respondents having pursued graduate studies.

**Table 3:** Respondents’ demographic profile.

Item	Demographics	Number of Respondents	Percentage (%)
Gender	male	180	47.2
	female	201	52.8
Age	18–29	187	49.1
	30–39	115	30.2
	40–49	53	13.9
	50–59	21	5.5
	≥60	5	1.3
Monthly income(¥)	<1000	136	35.7
	1000–3000	63	16.6
	3001–5000	34	8.9
	5001–8000	47	12.3
	8001–10000	47	12.3
	>10000	54	14.2
Educational level	Middle school or below	44	11.6
	High school	25	6.6
	College or university	156	40.9
	Postgraduate	156	40.9

#### 4.3.2 Factor analysis

We used SPSS 26 to conduct factor analysis to explore the model's reliability and validity. First, we conducted the Kisere-Meyere-Olkin (KMO) and Bartlett sphericity tests to assess whether the collected data were suitable for factor analysis. The KMO value for the scale was 0.933, which indicated that the collected sample size was suitable for factor analysis. Principal component analysis and the maximum variance method were employed to extract six factors from 24 items: aesthetics, creativity, eco-friendly, delicacy, attitude, and purchase intention. The overall explanatory power of the six factors reached 79.7 %, meeting the ideal criterion. Based on the criteria of factor analysis, we removed items with factor loadings below 0.6 and those that loaded onto incorrect factors. Ultimately, we obtained 18 items (Table 4).

**Table 4:** Rotated factor loading matrix.

	1	2	3	4	5	6
BEA1	0.832					
BEA2	0.797					
BEA4	0.688					
CREA1		0.827				
CREA2		0.785				
CREA4		0.610				
DEL2			0.735			
DEL3			0.685			
DEL4			0.807			
ECO2				0.809		
ECO3				0.733		
ECO4				0.838		
ATT1					0.662	
ATT3					0.770	
ATT4					0.709	
INT1						0.654
INT2						0.834
INT4						0.763

Extraction Method: Principal Component Analysis.

Rotation Method: Varimax with Kaiser Normalization.

a. Rotation converged in 6 iterations.

#### 4.3.3 Reliability and validity

We assessed the reliability and validity of the measurement scale. Reliability is related to the internal consistency of a scale. A scale with high reliability indicates that the items will produce similar results under similar conditions. Cronbach's alpha coefficient for each construct was higher than 0.7 (Table 5), indicating that each construct item measured the same fact. Validity is divided into convergent validity and discriminant validity. Convergent examines the degree to which different measures of a construct are consistent. The results show that the convergent validity for each construct is higher than 0.5 (average variance extracted (AVE)>0.5), indicating the scale's convergent validity. We also used the Fornell-Lacker criterion to measure the discriminant validity among the constructs. The results show that each construct correlates more with itself than with others, which indicates discriminant validity among the constructs. Therefore, our scale demonstrates sufficient reliability and validity.

**Table 5:** Reliability and validity.

Construct	Convergent Validity		Mean	Std. Deviation	Discriminant Validity					
	Cronbach's Alpha	AVE			INT	BEA	CREA	DEL	ECO	ATT
INT	0.836	0.568	4.885	1.129	**0.754**	0.532	0.533	0.510	0.571	0.685
BEA	0.875	0.600	5.453	1.218	0.532	**0.775**	0.603	0.683	0.474	0.607
CREA	0.838	0.557	5.176	1.105	0.533	0.603	**0.746**	0.687	0.462	0.596
DEL	0.879	0.554	5.396	1.113	0.510	0.683	0.687	**0.744**	0.518	0.641
ECO	0.852	0.631	5.112	1.085	0.571	0.474	0.462	0.518	**0.794**	0.645
ATT	0.885	0.511	4.964	1.050	0.685	0.607	0.596	0.641	0.645	**0.715**

#### 4.3.4 Regression analysis

We analyzed the relationships between the independent variables and the dependent variable through linear regression. First, we examined how beauty, creativity, delicacy, and eco-friendliness were related to attitudes toward Gejia batik. The results showed that all four independent variables were positively and significantly associated with attitudes (p < 0.001) (Table 6). Additionally, the variance inflation factor (VIF)<5 indicated no collinearity issues among the independent variables. R^2^ for the impact of the four predictor variables on attitudes is 0.587, which means that these four independent variables explain 58.7 % of the variance in the dependent variable, reaching a moderate level of explanation. We next conducted a regression analysis on the association between attitudes and purchase intention. The results showed a significant and positive relationship between attitudes and purchase intention (p < 0.001) (Table 6). Moreover, VIF<5 indicates no collinearity issues among the independent variables. The R^2^ value is 0.469, indicating that attitudes explain 46.9 % of the variance in purchase intention, reaching a moderate level of explanation. Finally, we examined how beauty, creativity, delicacy, and eco-friendliness were associated with purchase intention for Gejia batik. The results showed that except for delicacy (p = 0.596), the other three independent variables were positively and significantly associated with purchase intention (p < 0.001) (Table 6). Additionally, VIF<5 indicated no collinearity issues among the independent variables. R^2^ for the impact of the four predictor variables on attitudes is 0.442, which means that these four independent variables explain 44.2 % of the variance in the dependent variable, reaching a moderate level of explanation.

**Table 6:** Regression analysis.

DV	IV	Unstandardized Coefficients		Standardized Coefficients	Sig.	Collinearity Statistics		R^2^
		B	Std. E	Beta		Tolerance	VIF	
ATT	(Constant)	0.362	0.203		0.075			0.587
	BEA	0.164	0.041	0.190	0.000	0.488	2.051	
	CREA	0.163	0.045	0.172	0.000	0.486	2.056	
	DEL	0.190	0.050	0.201	0.000	0.396	2.525	
	ECO	0.360	0.039	0.372	0.000	0.693	1.443	
INT	(Constant)	1.231	0.204		0.000			0.469
	ATT	0.736	0.040	0.685	0.000	1.000	1.000	
INT	(Constant)	0.603	0.252		0.017			0.442
	BEA	0.193	0.051	0.209	0.000	0.488	2.051	
	CREA	0.227	0.056	0.222	0.000	0.486	2.056	
	DEL	0.033	0.062	0.032	0.596	0.396	2.525	
	ECO	0.368	0.048	0.353	0.000	0.693	1.443	

#### 4.3.5 Exploring the mediating role of attitude

Hayes' PROCESS macro for SPSS was employed (Model 4, sampling 5000 times) to test for the mediating relationship in the model. We used the simple mediator model for more than one predictor to examine the direct and indirect relationships in the model. The results are shown in Table 7. The score interval for the path of “beauty-attitude-intention” did not include 0 (CI: [0.032,0.131]), indicating that attitude significantly mediated the relationship between aesthetics and purchase intention, with a mediating score of 0.075. The relationship between aesthetics and purchase intention was also significant [0.023,0.213] after controlling for attitude. The coefficient for the direct relationship between beauty and purchase intention was 0.118. The score interval for the path of “creativity-attitude-intention” did not include 0 (CI: [0.027,0.134]), indicating that attitude significantly mediated the relationship between creativity and purchase intention, with a mediating score of 0.075. The relationship between creativity and purchase intention was also significant (CI: [0.047,0.256]) after controlling for attitude. The coefficient for the direct relationship between creativity and purchase intention was 0.151. The score interval for the path of “eco-friendly-attitude-intention” did not include 0 (CI: [0.112,0.237]), indicating that attitude significantly mediated the relationship between eco-friendly and purchase intention, with a mediating score of 0.166. The relationship between eco-friendly and purchase intention was also significant (CI: [0.104,0.299]) after controlling for attitude. The coefficient for the direct relationship between eco-friendly and purchase intention was 0.201. The path of “delicacy-attitude-intention” did not include 0 (CI: [0.039,0.147]), indicating that attitude significantly mediated the relationship between delicacy and purchase intention, with a mediating score of 0.088. Although the data did not support a direct relationship between delicacy and purchase intention, the indirect path indicated that the mediator variable (attitude) played a significant role in explaining the relationship. In this situation, attitude played a mediating role in each path.

**Table 7:** Mediating-effect analysis.

Path	Effect	Std.E	LLCI	ULCI
Direct effect: BEA→INT	0.118	0.048	0.023	0.213
Indirect effect: BEA→ATT→INT	0.075	0.025	0.033	0.132
Direct effect: CREA→INT	0.151	0.053	0.047	0.256
Indirect effect: CREA→ATT→INT	0.075	0.027	0.028	0.133
Direct effect: ECO→INT	0.201	0.049	0.104	0.299
Indirect effect: ECO→ATT→INT	0.166	0.032	0.109	0.235
Direct effect: DEL→INT	−0.055	0.059	−0.170	0.060
Indirect effect: DEL→ATT→INT	0.088	0.028	0.040	0.147

Note: Std. E = standard error.

#### 4.3.6 Hypothesis-testing results

Based on the regression and intermediary-relationship analyses, the path-coefficient results in Table 6, Table 7 can serve as a basis for testing all the hypotheses proposed in this study, and the results are presented in Table 8.

**Table 8:** Hypothesis-testing results.

Hypotheses	Sub-hypotheses	Path	Std.SE	P	Result
H1	H1a	Beauty is positively and significantly associated with consumers' attitudes.	0.041	0.000	Supported
	H1b	Beauty is positively and significantly associated with consumers' purchase intention.	0.051	0.000	Supported
	H1c	Consumers' attitude mediates the relationship between beauty and purchase intention.	0.025	0.000	Supported
H2	H2a	Creativity is positively and significantly associated with consumers' attitudes	0.045	0.000	Supported
	H2b	Creativity is positively and significantly associated with consumers' purchase intention.	0.056	0.000	Supported
	H2c	Consumers' attitude mediates the relationship between creativity and purchase intention.	0.027	0.001	Supported
H3	H3a	Delicacy is positively and significantly associated with consumers' attitudes.	0.050	0.000	Supported
	H3b	Delicacy is positively and significantly associated with consumers' purchase intention.	0.062	0.596	Not Supported
	H3c	Consumers' attitude mediates the relationship between delicacy and purchase intention(H3c).	0.032	0.001	Supported
H4	H4a	Eco-friendliness is positively and significantly associated with consumers' attitudes.	0.039	0.000	Supported
	H4b	Eco-friendliness is positively and significantly associated with consumers' purchase intention.	0.048	0.000	Supported
	H4c	Consumers' attitude mediates the relationship between eco-friendliness and purchase intention.	0.028	0.000	Supported

According to the hypothesis-testing results, as shown in Tables 8, it can be inferred that H1, H2, and H4 were supported, while H3 was partially supported. Based on the result presented in Table 8, we can conclude that beauty, creativity, and eco-friendliness in this model are positively and significantly associated with attitude and consumers’ purchase intention. Attitude mediates the relationship between these factors and purchase intention.

## 5 Discussion and implications

This study's findings provide valuable insights into the relationship between the beauty of Gejia batik products and consumers' purchase intention. A significant and positive relationship was observed between beauty and consumers' purchase intention. This result is consistent with Liu's [46] and Yen's [48] findings, which emphasize the importance of aesthetic appeal for consumer behavior. The beauty of Gejia batik products can be attributed to various factors, including intricate patterns, vibrant colors, and skilled craftsmanship. These aesthetic qualities not only enhance the visual appeal of the products but also evoke a sense of admiration and appreciation among consumers. The positive relationship between the beauty of Gejia batik and consumers' purchase intention has important implications for both batik manufacturers and marketers. Manufacturers should focus on creating visually appealing designs, investing in skilled craftsmanship, and using high-quality materials to enhance the beauty of Gejia batik products. Thus, they can attract more consumers and increase their purchase intention.

The findings also reveal a significant and positive relationship between the creativity of Gejia batik products and consumers' purchase intention. This finding aligns with prior research that has emphasized the importance of creativity in consumer behavior [46]. The creativity of Gejia batik products can be attributed to various factors, including innovative design elements, unique patterns, and the incorporation of contemporary themes. These creative aspects can not only enhance the visual appeal of the products but can also evoke a sense of novelty and uniqueness among consumers. Therefore, manufacturers should focus on fostering a culture of creativity within their design teams, encouraging experimentation, and pushing the boundaries of traditional Gejia batik designs. Thus, they can create products that stand out in the market and attract consumers' attention.

Based on the results of this study, the eco-friendliness of Gejia batik products has the most significant and positive relationship with consumers' purchase intention. This indicates that the extent to which Gejia batik products are environmentally friendly plays a crucial role in consumers' decision to purchase these products. This finding aligns with prior research that has emphasized the growing importance of sustainability and eco-consciousness in consumer behavior [49]. The findings have important implications for both batik manufacturers and marketers. Manufacturers should continue to prioritize sustainability in their production processes and explore innovative ways to further reduce the environmental footprint of Gejia batik, such as using eco-friendly materials and minimizing waste generation. Thus, manufacturers can attract environmentally conscious consumers and differentiate themselves in the market.

Therefore, in the product development of Gejia batik, it is important to fully consider attributes such as beauty, creativity, and eco-friendliness. This can be achieved by focusing on creating visually appealing designs, promoting artistic creativity, and ensuring eco-friendly production processes and materials.

In addition, consumer attitude is significantly associated with purchase intention, consistent with the research findings in many consumer domains [50,51]. Consumers' attitudes toward a product are associated with their purchase intention, and the more positive their attitude, the stronger their intention to engage in consumption behavior. Kraus [52] pointed out that attitude was relatively enduring and stable as a measurable psychological construct. It has a positive association with the generation of behavior. Therefore, batik enterprises should prioritize efforts to cultivate a positive attitude toward their products. This can be achieved through effective marketing and promotional strategies that highlight the unique qualities of Gejia batik, such as its cultural significance, craftsmanship, and sustainability. By creating a positive perception of the product, batik enterprises can increase sales and establish a loyal consumer base.

However, the delicacy of batik products does not positively impact consumers' purchase intention, which is inconsistent with Liu's [46] findings. For batik products, the delicacy of batik primarily depends on the patterns' complexity and lines' thickness. In modern society, aesthetic trends constantly evolve, and people prefer simple, modern, and abstract art styles. Therefore, batik enterprises should consider incorporating contemporary design elements and patterns that align with current aesthetic preferences to attract a broader consumer base.

The mediating role of consumer attitudes in the relationships between batik qualia factors (beauty, creativity, eco-friendliness) and consumers' purchasing intention further explains the relationships between qualia factors (beauty, creativity, eco-friendliness) and consumers' willingness to purchase. In other words, the influence of batik products' qualia factors (beauty, creativity, and eco-friendliness) does not directly affect consumers' purchasing intention but is transmitted through the intermediate link of consumers' product attitudes. As a strongly endogenous variable, attitude often serves as a mediating variable in consumer behavior. Therefore, businesses can influence consumers' attitudes toward products through marketing or promotional means, thereby changing their purchasing intentions.

### 5.1 Theoretical implications

By investigating consumers' purchase intentions for Gejia batik, our study contributes to the existing literature in several ways. First, it fills a research gap in the field of consumption studies, as previous research has primarily focused on other consumer goods and industries. This study deepens the understanding of market and consumer behavior, specifically in the context of Gejia batik products.

Second, our study validates and expands existing consumer-behavior theories by examining the relationship between the qualia (beauty, creativity, and eco-friendliness) of batik products and consumers' purchasing intention. This analysis provides empirical evidence for the role of sensory experiences, emotional connections, and value perceptions in consumer decision-making processes. Thus, our study contributes to the theoretical development of consumer behavior theories.

Last, examining purchasing intention for ICHPs, such as Gejia batik, holds significant importance in the field of cultural economics. Analyzing consumers' perceptions of beauty, creativity, eco-friendliness, attitude, and purchase intention for ICHPs can reveal the economic potential of these products and provide theoretical support for the sustainable development of the cultural industry.

### 5.2 Practical implications

The results of our study have practical implications for various stakeholders involved in the production, marketing, and preservation of Gejia batik products.

For batik manufacturers and designers, understanding consumers' demand for qualia and purchasing intention for batik products can guide product development and market positioning. By improving the aesthetics, creativity, and eco-friendly material of the products, manufacturers can better meet consumers' expectations for high quality and uniqueness. This, in turn, can enhance consumers' purchasing intention and loyalty toward batik products.

Moreover, our study provides insights into effective marketing and communication strategies for batik products. Emphasizing the unique features, creativity, and eco-friendliness of batik products in promotional activities can attract more consumers and increase their purchasing intention. This knowledge can guide businesses in designing marketing campaigns that effectively highlight the advantages and uniqueness of batik products.

Furthermore, our findings can inform government agencies and practitioners involved in the preservation and development of batik craftsmanship. Understanding consumers' requirements for the qualia of batik products and their purchasing intention can guide efforts in the inheritance and promotion of batik craftsmanship. This knowledge can contribute to the sustainability of traditional crafts and cultural industry as a whole.

Last, enhancing the qualia (beauty, creativity, and eco-friendliness) of batik products has the potential to attract cultural tourists. By improving the sensory experiences and emotional connections associated with batik products, it becomes possible to attract more tourists to experience and purchase them. This, in turn, promotes the development of local cultural tourism, enhances the visibility and attractiveness of the destination, and contributes to the local economy.

### 5.3 Limitations and future research

As with all research, this study has some limitations. First, the sample for this study was obtained through convenience sampling. Convenience sampling is a non-probability sampling method that is based on the researcher's convenience and accessibility in selecting the sample. Moreover, the sample comprises mainly young people who are 21–29 years old, who accounted for 49.1 % of the total sample size. They are not typical buyers of batik products. Additionally, most of the respondents have undergraduate and postgraduate degrees, and this group is not representative. Because convenience sampling, sample age, and education distribution do not guarantee the sample's representativeness, the research results may not be generalizable to the entire target population. Therefore, in future, we can adopt the random sampling method, increase the sample size, and standardize the distribution of observations as to make the research results more representative. Second, this study only included the qualia factors of batik products as independent variables to explain consumers' behavioral intentions, and the independent variables only explained 44.2 % of the variance in purchase intentions. In statistical terms, the explanatory power of these independent variables on the dependent variable reached only a moderate level, suggesting that other variables may be more influential in affecting purchase intentions. Therefore, in future research, it would be beneficial to include other variables, such as cultural identity, product price, subjective norms, etc., to conduct further investigations. Third, according to the results, eco-friendliness has the most significant impact on cunsumers among all the variables, and this conclusion may be exaggerated. There are two possible reasons for this. First, most of the participants involved in this study are highly educated, and individuals with higher education levels tend to have higher environmental demands. Second, respondents may be influenced by social expectations when answering questions. If respondents believe that eco-friendly responses are more socially desirable, they may be inclined to report more eco-friendly behaviors. In future studies, more control variables and different study designs could be considered to verify the robustness of the results. Last, this study focuses on consumers' purchase intentions, and there can often be a discrepancy between behavioral intentions and actual behavior. Consumers' behavioral intentions do not always accurately predict their actual behavior. Even if consumers intend to purchase a particular product or take a specific action, other factors can influence or alter their actual purchasing behavior. Therefore, it would be beneficial to investigate other factors in consumers' purchasing behavior regardingGejia batik products in future research.

## Ethics statement

This study was conducted after receiving approval and permission from the Jawatankuasa Etika Penyelidikan Manusia Universiti Sains Malaysia (code: USM/JEPeM/PP/23020174), Because the data for this study were mainly collected through a questionnaire, it was not possible for everyone to fill in an informed-consent form, such as when an interview is used. Therefore, to simplify the process, we set an informed consent option at the beginning of the questionnaire, and those who checked this option indicated that they granted informed consent. Moreover, we confirmed that informed consent was obtained from all the participants in our survey.

## Funding

This research was funded by the General Project of Philosophy and Social Sciences for Universities in Jiangsu Province, grant number (2022SJYB2012).

## Data availability statement

Due to ethical review requirements, research-related data are not kept in publicly available repositories, and all research data is accessible only to researchers. Anyone who needs data to reproduce research results can email the author to obtain the data.

## CRediT authorship contribution statement

**Xizhen Li:** Writing – review & editing, Writing – original draft, Visualization, Validation, Supervision, Software, Resources, Project administration, Methodology, Investigation, Funding acquisition, Formal analysis, Data curation, Conceptualization. **Nurul Hanim Romainoor:** Writing – review & editing, Supervision. **Zhiqin Sun:** Investigation, Data curation.

## Declaration of competing interest

The authors declare that they have no known competing financial interests or personal relationships that could have appeared to influence the work reported in this paper.

## Figures and Tables

**Table A:** Independent-sample *t*-test results for each item

Item	*t*-test for Equality of Means				group	N	Mean	Std. Deviation
	t	df	Sig. (2-tailed)	Mean Difference				
BEA1	−6.255	28	0.000	−2.000	low	15	4.867	1.187
					high	15	6.867	0.352
BEA 2	−6.975	28	0.000	−1.933	low	15	4.867	0.990
					high	15	6.800	0.414
BEA 3	−6.513	28	0.000	−2.133	low	15	4.600	1.121
					high	15	6.733	0.594
BEA 4	−8.799	28	0.000	−2.733	low	15	3.933	1.100
					high	15	6.667	0.488
CREA1	−9.691	35	0.000	−2.301	low	19	4.421	0.902
					high	18	6.722	0.461
CREA 2	−7.909	35	0.000	−2.026	low	19	4.474	0.964
					high	18	6.500	0.514
CREA 3	−8.658	35	0.000	−2.202	low	19	4.632	1.012
					high	18	6.833	0.383
CREA 4	−9.446	35	0.000	−2.561	low	19	4.105	1.049
					high	18	6.667	0.485
DEL1	−9.648	30	0.000	−2.750	low	16	3.875	1.025
					high	16	6.625	0.500
DEL2	−11.560	30	0.000	−2.625	low	16	4.313	0.873
					high	16	6.938	0.250
DEL3	−12.632	30	0.000	−2.750	low	16	4.188	0.834
					high	16	6.938	0.250
DEL4	−7.814	30	0.000	−2.125	low	16	4.875	1.088
					high	16	7.000	0.000
ECO1	−9.081	33	0.000	−2.383	low	15	4.267	0.884
					high	20	6.650	0.671
ECO2	−12.961	33	0.000	−2.800	low	15	3.800	0.775
					high	20	6.600	0.503
ECO3	−8.520	33	0.000	−2.133	low	15	4.267	0.704
					high	20	6.400	0.754
ECO4	−9.212	33	0.000	−2.400	low	15	4.200	1.014
					high	20	6.600	0.503
ATT1	−8.258	37	0.000	−1.939	low	17	4.471	0.874
					high	22	6.409	0.590
ATT2	−12.478	37	0.000	−2.513	low	17	3.941	0.748
					high	22	6.455	0.510
ATT3	−16.176	37	0.000	−2.468	low	17	3.941	0.429
					high	22	6.409	0.503
ATT4	−9.707	37	0.000	−2.441	low	17	4.059	1.029
					high	22	6.500	0.512
INT1	−8.128	32	0.000	−2.863	low	19	3.737	1.284
					high	15	6.600	0.507
INT 2	−6.080	32	0.000	−2.046	low	19	4.421	1.170
					high	15	6.467	0.640
INT 3	−9.584	32	0.000	−2.653	low	19	3.947	0.970
	−10.271	28.260	0.000	−2.653	high	15	6.600	0.507
INT 4	−7.482	32	0.000	−2.400	low	19	4.000	1.155
	−8.122	25.883	0.000	−2.400	high	15	6.400	0.507

**Table B:** Skewness and kurtosis for normality test

	N	Minimum	Maximum	Mean	Std. Deviation	Skewness		Kurtosis	
	Statistic	Statistic	Statistic	Statistic	Statistic	Statistic	Std. Error	Statistic	Std. Error
AES1	381	1.00	7.00	5.556	1.316	−1.196	0.125	1.211	0.249
AES2	381	1.00	7.00	5.567	1.327	−1.183	0.125	1.129	0.249
AES4	381	1.00	7.00	5.236	1.437	−0.853	0.125	0.177	0.249
CREA1	381	1.00	7.00	5.131	1.291	−0.755	0.125	0.368	0.249
CREA2	381	1.00	7.00	5.278	1.166	−0.757	0.125	0.438	0.249
CREA4	381	1.00	7.00	5.118	1.349	−0.623	0.125	−0.051	0.249
DEL2	381	1.00	7.00	5.388	1.242	−0.745	0.125	0.472	0.249
DEL3	381	1.00	7.00	5.304	1.268	−0.486	0.125	−0.039	0.249
DEL4	381	1.00	7.00	5.496	1.213	−0.649	0.125	−0.149	0.249
GRE2	381	1.00	7.00	5.029	1.274	−0.430	0.125	−0.303	0.249
GRE3	381	1.00	7.00	5.121	1.217	−0.418	0.125	−0.393	0.249
GRE4	381	1.00	7.00	5.186	1.216	−0.423	0.125	−0.338	0.249
ATT1	381	1.00	7.00	5.118	1.071	−0.625	0.125	0.176	0.249
ATT3	381	1.00	7.00	4.738	1.227	−0.056	0.125	−0.493	0.249
ATT4	381	2.00	7.00	5.037	1.192	−0.400	0.125	−0.306	0.249
INT1	381	1.00	7.00	4.890	1.361	−0.650	0.125	0.038	0.249
INT2	381	1.00	7.00	4.945	1.269	−0.712	0.125	0.553	0.249
INT4	381	1.00	7.00	4.822	1.269	−0.376	0.125	−0.116	0.249
