# Supplementary material for: Factors in consumers' purchase intention for Gejia batik
Source: Heliyon. 2023 Nov 30;10(1):e23085. doi: 10.1016/j.heliyon.2023.e23085 (PMC10756975; doi:10.1016/j.heliyon.2023.e23085)

**Factors Influencing Consumers’ Purchase Intention of Gejia Batik**

**Questionnaire**

Dear Participant,

Thank you for taking the time to participate in this research study. Your input is valuable and will contribute to the advancement of knowledge in Intangible cultural heritage product marketing

Before you proceed with the questionnaire, we kindly ask you to read the following information carefully.

**Informed Consent for Participation in Survey**

**Title:** [Factors Influencing Consumers’ Purchase Intention of Gejia Batik]

**Researcher:** [LI XIZHEN]

**Contact Information:** [379076203@qq.com / 15161986239]

**Introduction:**

You are invited to participate in a research survey conducted by Li Xizhen, as part of a study on batik product purchase intention. Your voluntary participation in this survey is greatly appreciated. Before you decide to participate, we kindly ask you to read this consent form and understand the information provided. If you have any questions or concerns, please feel free to contact us using the provided contact information.

**Purpose of the Study:**

The purpose of this survey is to understand consumers' attitudes and intentions toward purchasing batik products. By participating in this survey, you will help us gain valuable insights into consumer preferences and contribute to the enhancement of batik product offerings.

**Procedures:**

If you agree to participate in this survey, you will be asked to answer a series of questions related to your preferences, purchasing behaviors, and attitudes toward batik products. The estimated time to complete the survey is approximately 5-10 minutes.

**Voluntary Participation:**

Your participation in this survey is entirely voluntary. You have the right to refuse to participate or withdraw your participation at any time without facing any negative consequences. Your decision to participate or not will not affect your relationship with us.

**Confidentiality and Anonymity:**

Your responses will be kept confidential to the extent permitted by law. Any information that is obtained during the survey will be anonymized, and no personally identifiable information will be linked to your responses. Only the researchers involved in this study will have access to the data, and all data will be stored securely.

**Risks and Benefits:**

There are no foreseeable risks associated with participating in this survey. Your participation will be beneficial in providing valuable insights that may contribute to the improvement of batik product offerings in the market.

**Data Usage:**

The data collected in this survey will be used for research purposes only. The findings may be used in research reports or publications; however, your individual responses will remain anonymous.

**Contact Information:**

If you have any questions or concerns about this survey or the research in general, please feel free to contact [Li Xizhen] at [379076203@qq.com / 15161986239].

By proceeding with this survey, you indicate that you are at least 18 years old and have read and understood the information provided in this consent form. Your decision to participate is voluntary, and you may withdraw your consent at any time during the survey.

A √ in the box where I agree indicates that you have read and understood the information provided in this consent form and voluntarily agree to participate in the survey.

- **I agree to participate in the survey.**
- **I do not agree to participate in the survey.**

If you volunteer for this study, you can start answering.

Date: 08/06/2023

**Section A Demographic Profile**

Please tick （√） at the relevant box.

1. Sex: Male female
2. Age: < 18 18-29 30-39

40-49 50-59 >=60

1. What is your educational level?

Middle school or below

High school

College or university

Postgraduate

1. What's your monthly income now?

<1000￥ 1000-3000￥ 3001-5000￥

5001-8000￥ 8001-10000 ￥ >10000￥

1. The Selected Batik Products for Testing(Each questionnaire included a picture to test)

| Category | Num-  ber | Image | Category | Num-  ber | Image |
| --- | --- | --- | --- | --- | --- |
| Decorative picture | 1 | 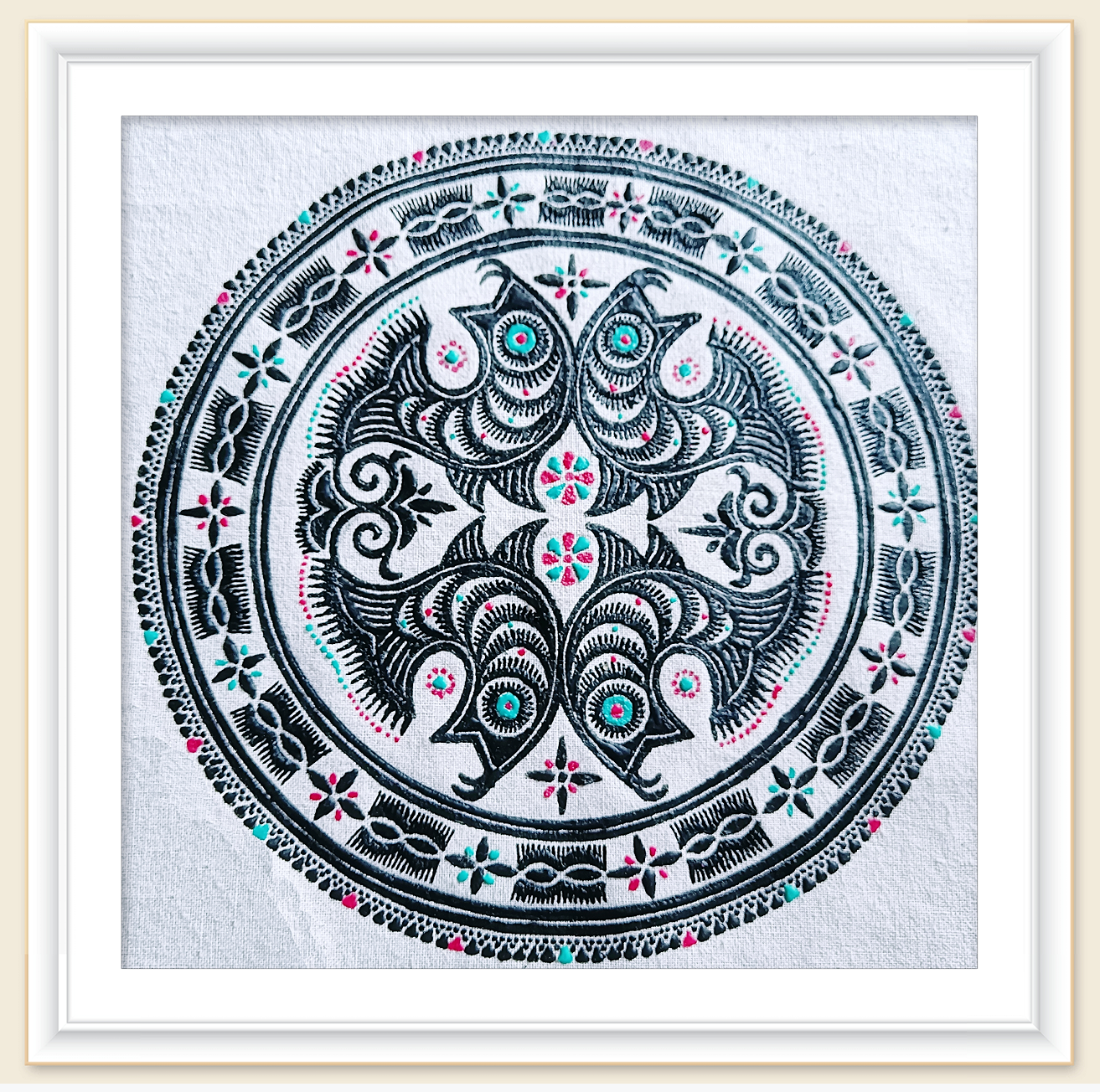 | Clothing | 9 | 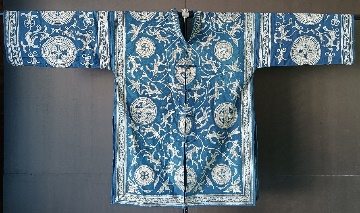 |
|  | 2 | 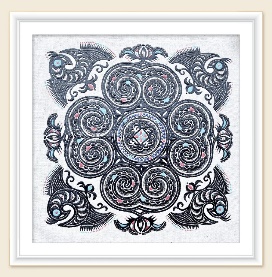 |  | 10 | 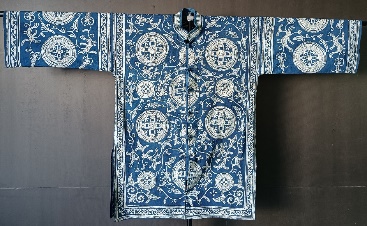 |
|  | 3 | 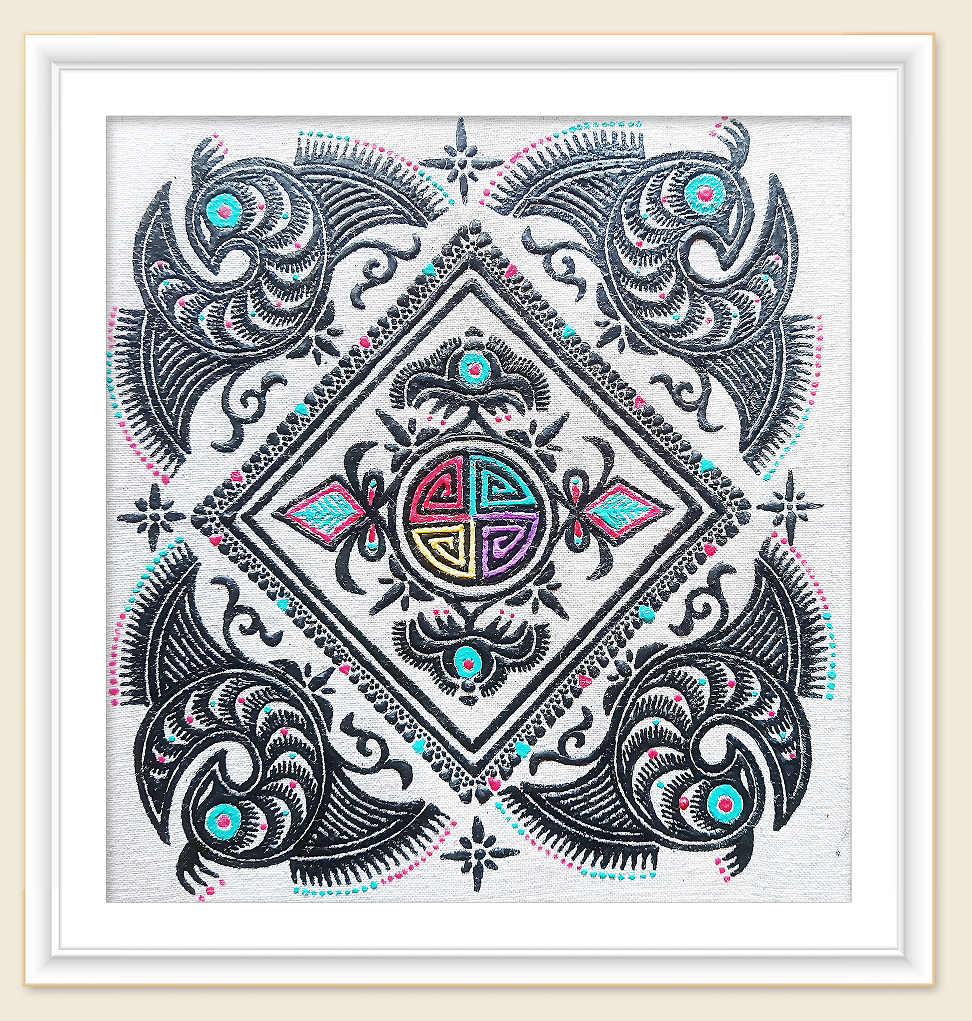 |  | 11 | 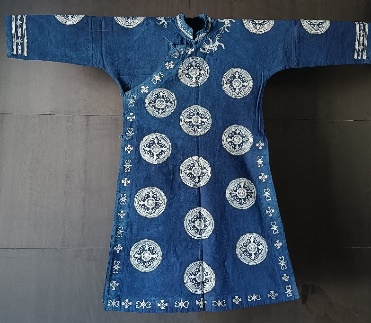 |
|  | 4 | 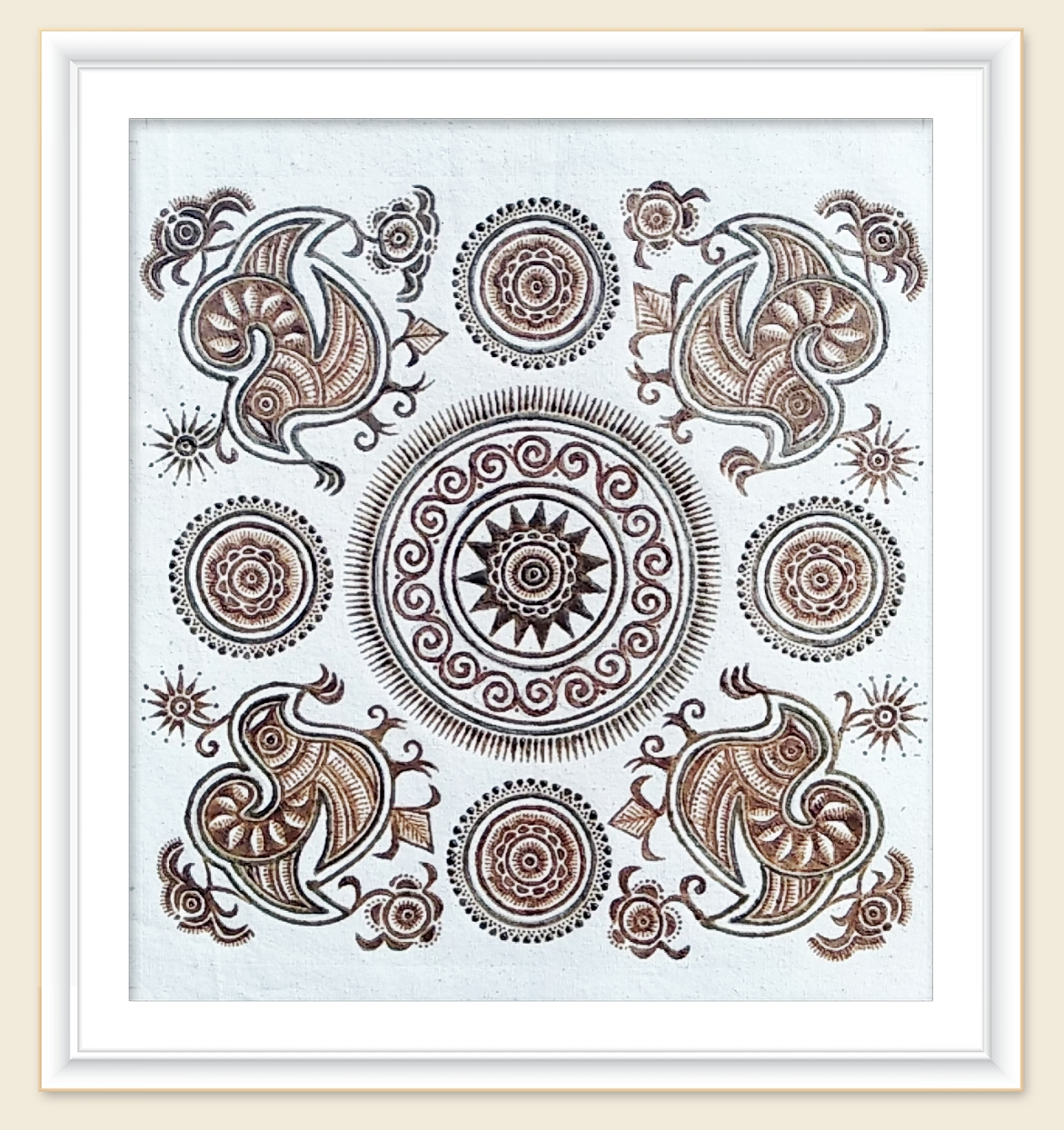 | Baby carriers | 12 | 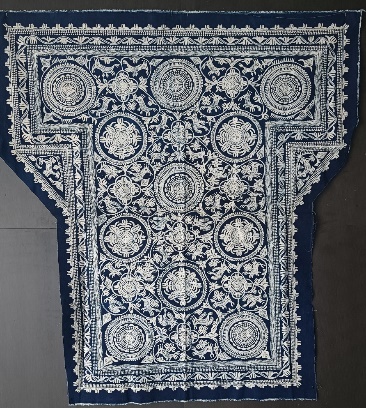 |
|  | 5 | 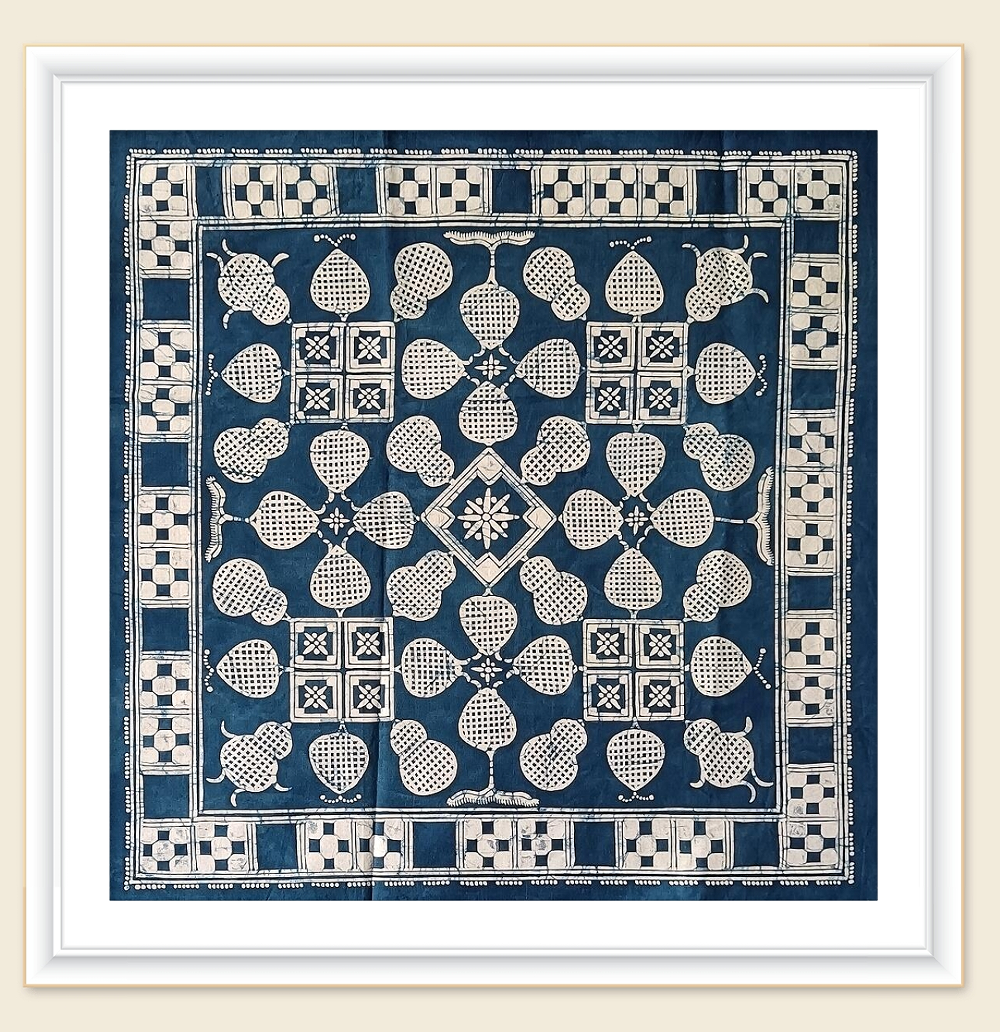 | Table cloth | 13 | 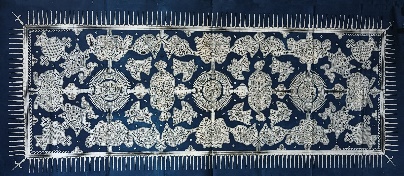 |
| Throw pillow | 6 | 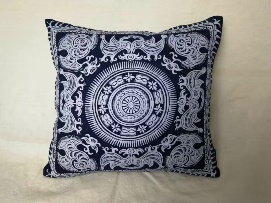 | Bag | 14 | 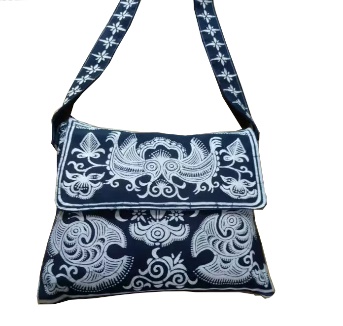 |
|  | 7 | 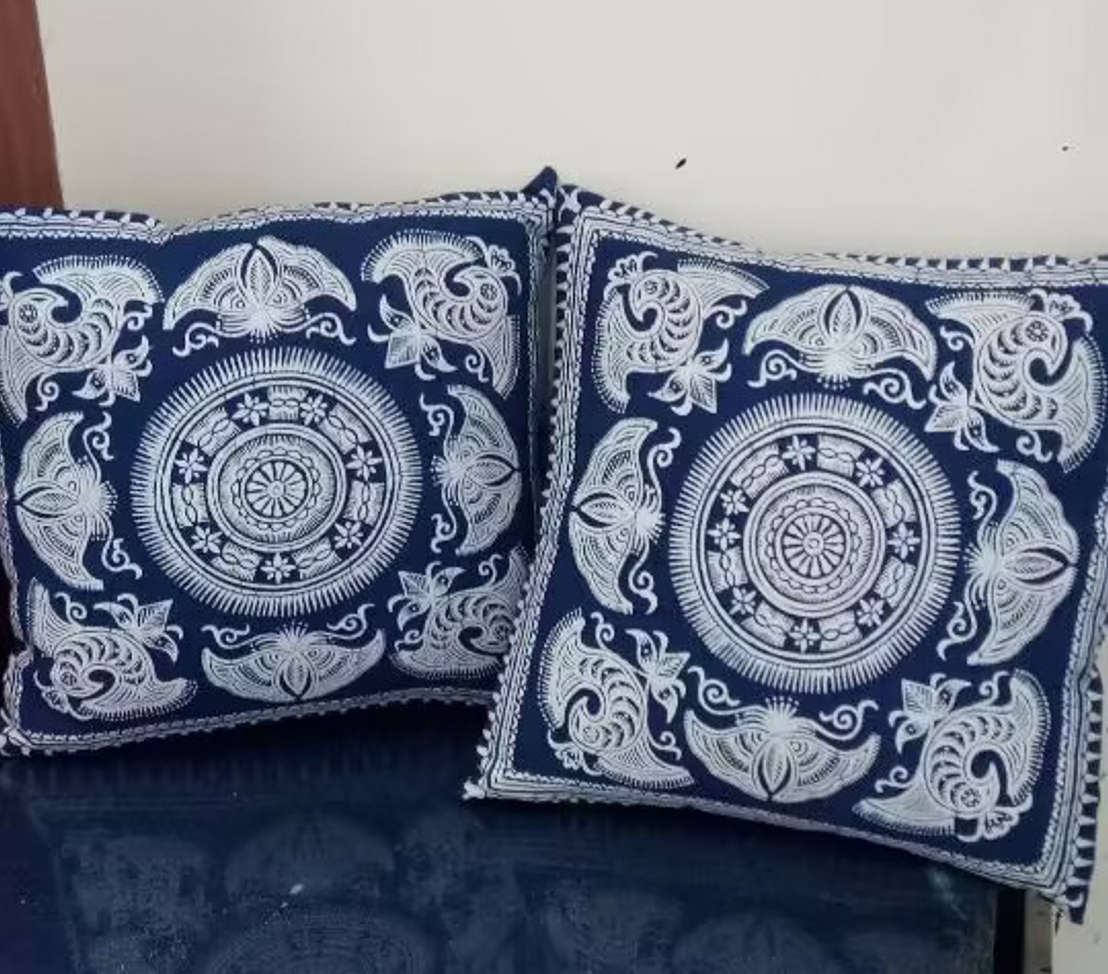 |  | 15 | 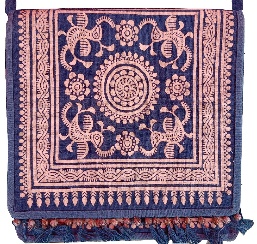 |
| Lumbar pillow | 8 | 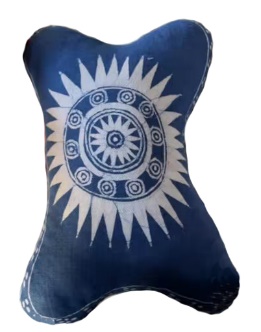 | Umbrella | 16 | 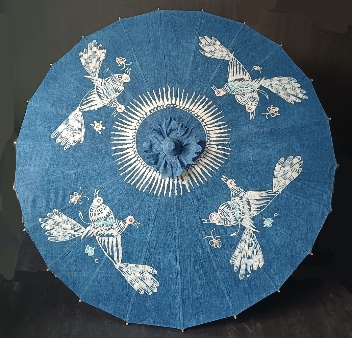 |

**Section B Choosing your innermost thoughts according to the description**

(Please tick [√] in the relevant box)

1. This Gejia batik product has aesthetic appeal.


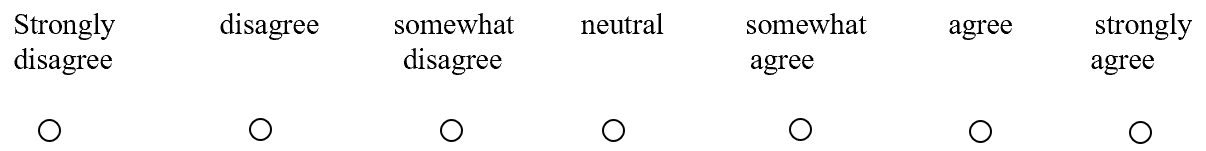


1. The color combination of this Gejia batik product is harmonious.


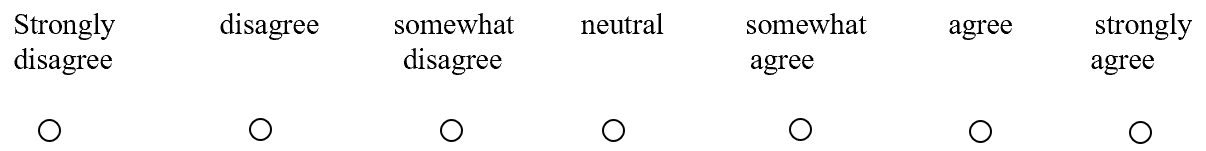


1. This batik product has a beautiful pattern


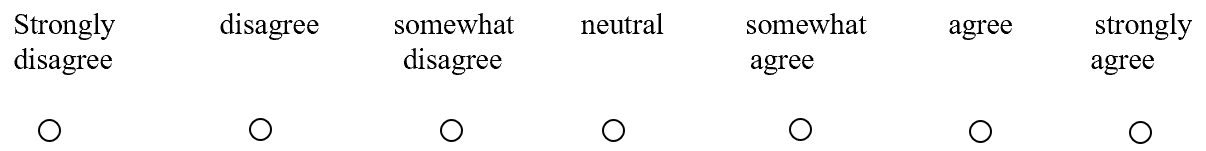


1. This Gejia batik product is visually pleasing.


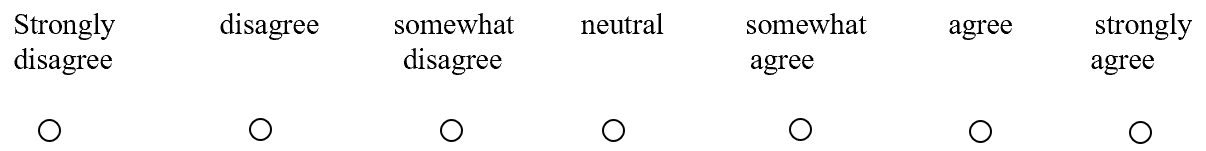


1. This Gejia batik is a creative product.


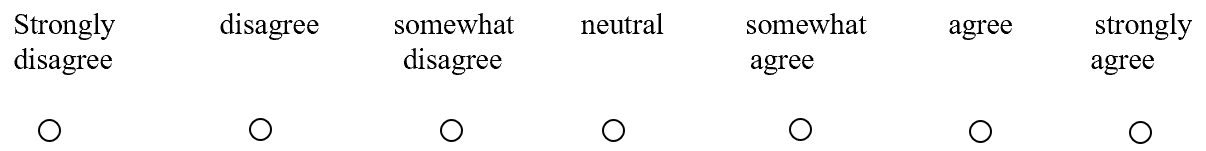


1. This Gejia batik product has well-designed.


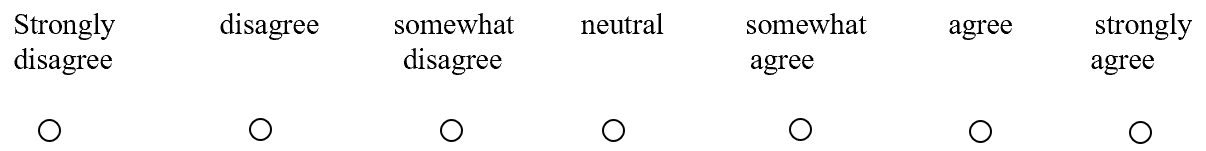


1. This Gejia Batik product possesses a certain level of artistic quality.


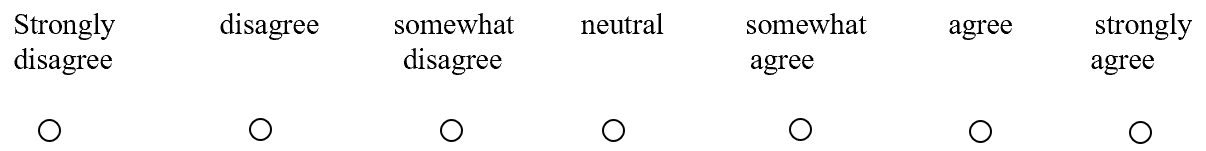


1. This Gejia batik product evokes a sense of novelty.


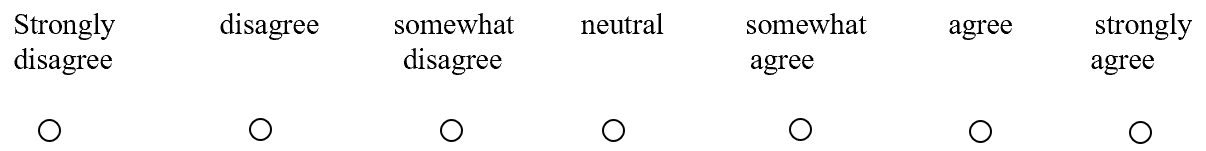


1. This Gejia batik product is exceptionally exquisite.


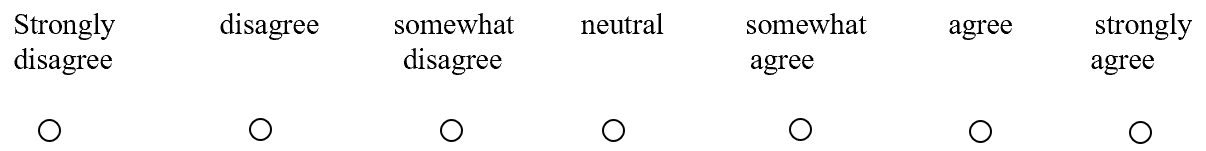


1. This Gejia batik is finely crafted.


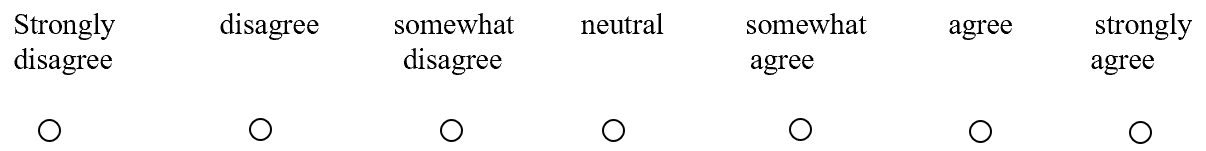


1. This Gejia batik product has a high level of craftsmanship and quality.


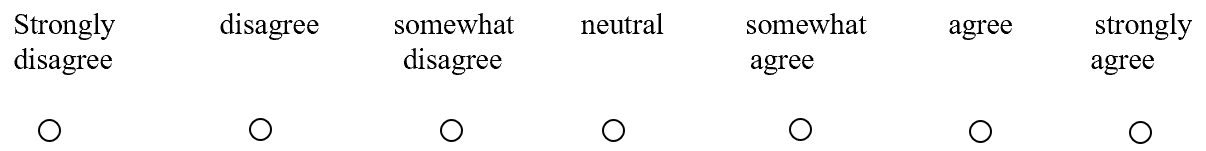


1. This Gejia batik was drawn with fine lines.


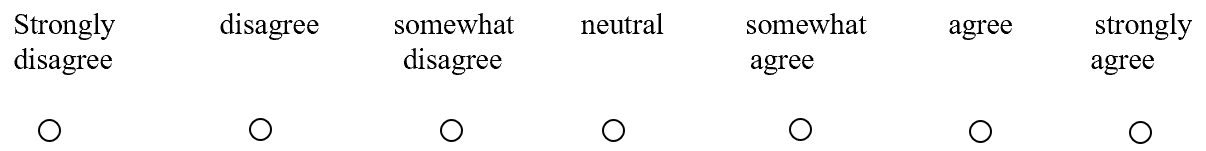


1. I think the fabric of Gejia Batik is comfortable, healthy, and consumer-friendly.


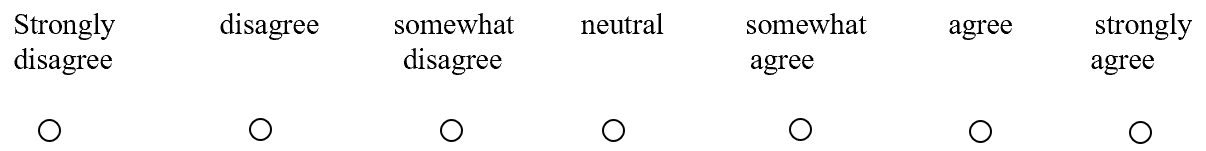


1. I think Gejia batik products have a minimal impact on the natural environment during the manufacturing process.


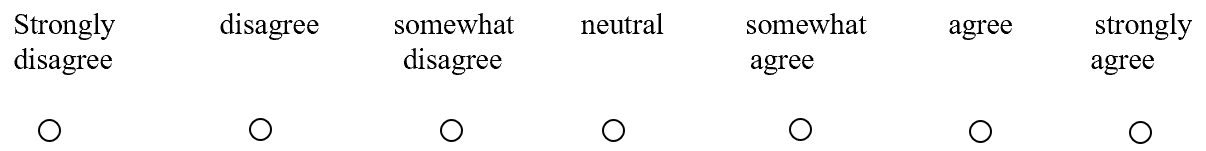


1. I believe that the fuel used in the production process of Gejia batik is safe and harmless to human health.


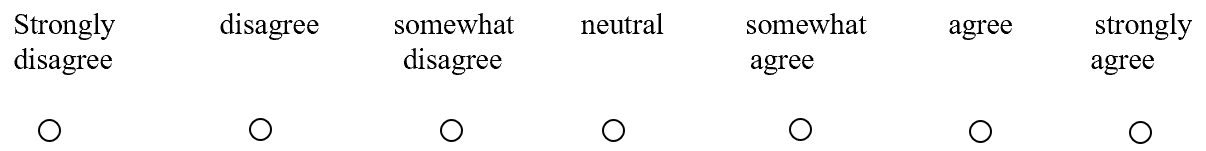


1. I believe that Gejia Batik is environmentally friendly in terms of the materials used in its production process.


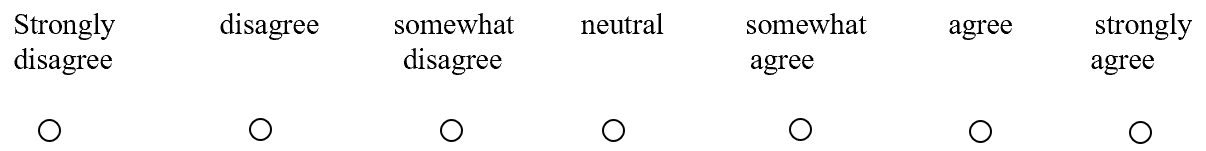


1. I think Gejia Batik is excellent.


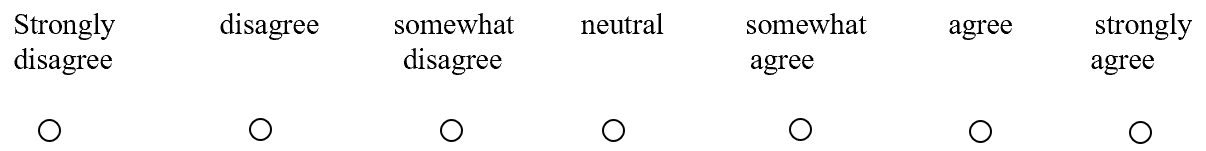


1. I like Gejia batik products


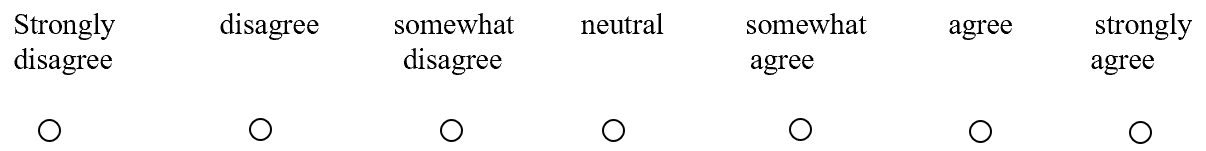


1. I prefer Gejia batik products over other batik products.


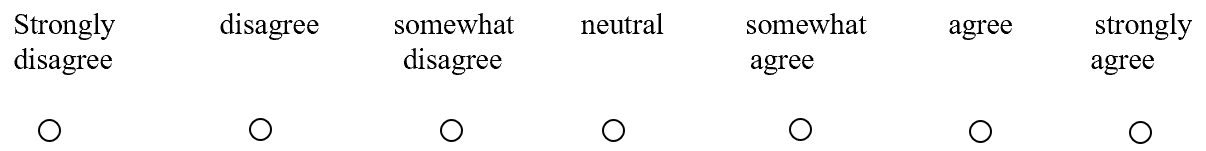


1. I think Gejia Batik products are worth buying.


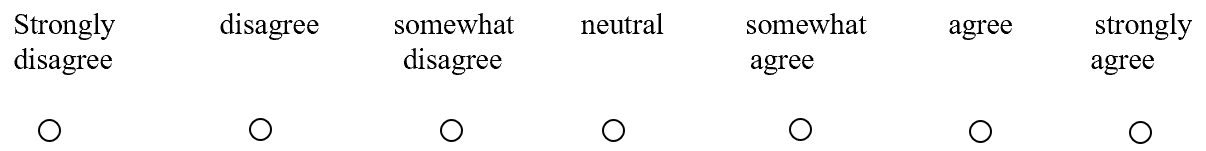


1. I will consider purchasing Gejia Batik products.


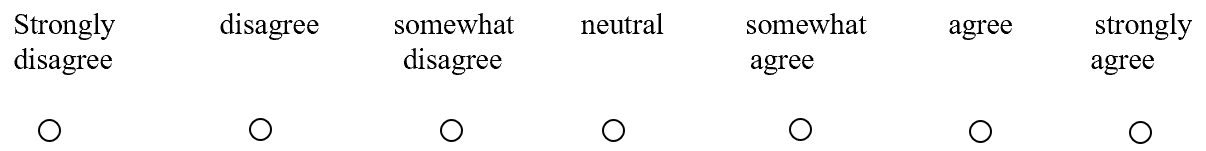


1. I am very likely to buy Gejia Batik products in the future.


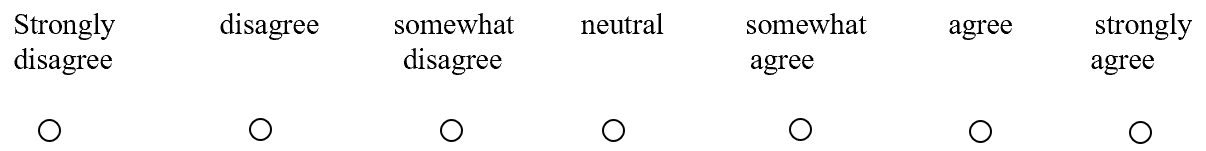


1. I will recommend others to purchase Gejia Batik.


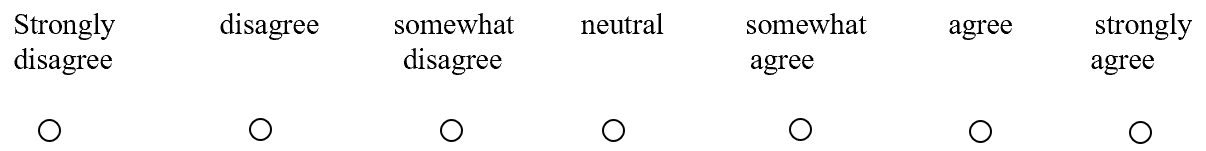


1. When encountering similar products, I would prioritize considering purchasing Gejia Batik products.


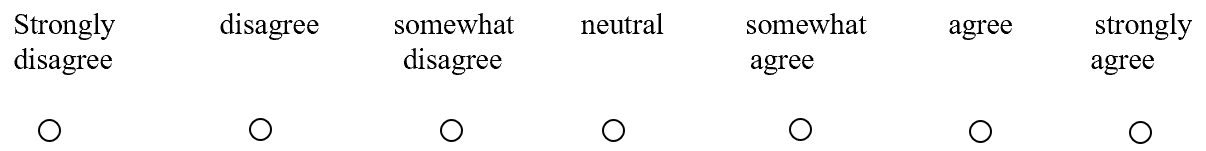

Supplement: Multimedia component 1 [file mmc1.docx]
